# Correlation between orthostatic intolerance in children and levels of ACE2-Ang(1-7)-Mas axis and vitamin D

**DOI:** 10.3389/fped.2025.1585032

**Published:** 2025-05-16

**Authors:** Yao Xiao, Jinzhi Wu, Jin Wang, Tingting He, Xiangyu Dong

**Affiliations:** ^1^ Department of Pediatrics, The Third Hospital of Changsha (Changsha Hospital Affiliated to Hunan University), Changsha, Hunan, China; ^2^ Department of Pediatrics, KaiLi First People's Hospital, KaiLi, Guizhou, China; ^3^ Department of Pediatrics, Lanzhou University Second Hospital, Lanzhou, Gansu, China

**Keywords:** orthostatic intolerance (OI), angiotensin-converting enzyme 2 (ACE2), angiotensin (1-7), vitamin D, 25-hydroxylase

## Abstract

**Objective:**

To explore the correlation between orthostatic intolerance in children and levels of the ACE2-Ang(1-7)-Mas axis and vitamin D.

**Methods:**

Blood samples were collected from 84 children with orthostatic intolerance and 307 healthy controls. After matching for age and sex, 84 children from each group were studied. The orthostatic intolerance group was divided into vasovagal syncope (*n* = 51) and postural orthostatic tachycardia syndrome (*n* = 33). Fasting blood samples were analyzed for 25(OH)D, ACE2, Ang(1-7), and hydroxylases using ELISA.

**Results:**

(1) The orthostatic intolerance group had significantly lower levels of ACE2, Ang(1-7), 25(OH)D, and hydroxylases compared to controls (*P* < 0.05). (2) No sex differences in biomarker levels were found in the orthostatic intolerance group (*P* > 0.05), but boys in the control group had higher 25(OH)D levels (*P* < 0.001). (3) No significant differences between the two intolerance subgroups (*p* > 0.05). (4) Logistic regression showed lower levels of 25(OH)D, 25-hydroxylase, and Ang(1-7) correlated with higher orthostatic intolerance incidence. (5) Ang(1-7) levels of 19.39 ng/ml provided 86.9% sensitivity and 61.9% specificity for diagnosis.

**Conclusion:**

Reduced levels of Ang(1-7)/ACE2, 25(OH)D, and 25-hydroxylase are linked to orthostatic intolerance in children, highlighting vitamin D deficiency's role and suggesting Ang(1-7) and ACE2 as potential biomarkers. Sex does not significantly affect these biomarker levels.

## 1 Background

Neurally mediated syncope (NMS) is the leading cause of syncope in children in China, about 20%–30% of children and adolescents aged 5–18 years have experienced at least one episode of syncope (1). Orthostatic intolerance (OI) is a common type of NMS in children. It is often manifested by fainting, dizziness, fatigue, blurred vision, abdominal discomfort, and other clinical symptoms, especially during moments of mental stress, prolonged standing, or changes in body position (2).

OI can be classified hemodynamically into four main types: vasovagal syncope (VVS), postural tachycardia syndrome (POTS), orthostatic hypotension (OH), and orthostatic hypertension (OHT), with VVS and POTS being the most common (2). In recent years, the pathological mechanism of OI has not been fully elucidated, which brings challenges to clinical diagnosis and treatment.

Recent studies have found that vitamin D, a fat-soluble vitamin, plays a significant regulatory role in the cardiovascular system in addition to its traditional function in regulating calcium and phosphate metabolism (3, 4). Vitamin D metabolism is regulated by three enzymes: 25-hydroxylase, which converts cholecalciferol (VD3) to 25(OH)D; 1α-hydroxylase, which activates it to 1,25(OH)₂D; and 24-hydroxylase, which generates inactive metabolites (5). The balance of these enzymes determines active vitamin D levels. Reduced 1α-hydroxylase activity or excessive 24-hydroxylase expression can lower active vitamin D levels. In deficiency, the body compensates by upregulating 1α-hydroxylase, though this mechanism may be insufficient in certain pathological conditions (5, 6). Vitamin D exerts its cardiovascular protective effects by binding to the vitamin D receptor (VDR), influencing the activity of the autonomic nervous system (7), and regulating the balance of the renin-angiotensin-aldosterone system (RAAS) (8, 9). However, the specific mechanisms underlying the role of vitamin D in the occurrence and development of OI remain unclear.

Meanwhile, the imbalance of the angiotensin-converting enzyme 2 (ACE2)-angiotensin (1-7) [Ang(1-7)]-Mas receptor axis has been implicated in various cardiovascular diseases (10). This pathway protects endothelial function by promoting vasodilation and counteracting the vasoconstrictive effects of Ang II (11, 12). Existing studies suggest that vitamin D deficiency may exacerbate the imbalance of the Renin-angiotensin-aldosterone system (RAAS) by downregulating ACE2 expression, thereby affecting cardiovascular function (13–15). However, whether children with OI exhibit abnormalities in the ACE2-Ang(1-7)-Mas axis, and the specific mechanisms through which this axis contributes to the pathogenesis of OI, remain inadequately studied.

Therefore, the present study hypothesizes that children with OI have abnormal activity of vitamin D metabolic enzymes, including 24-hydroxylase, 25-hydroxylase, and 1α-hydroxylase, leading to vitamin D deficiency. This deficiency may inhibit the ACE2-Ang(1-7)-Mas axis, resulting in elevated Ang II levels, which could contribute to autonomic dysregulation and vascular dysfunction.

## 2 Data and methods

### 2.1 Study subjects

This study collected whole blood specimens from 84 pediatric patients diagnosed with OI through Head-up test (HUT) or Head-up tilt test (HUTT) at the Lanzhou university second hospital from December 2019 to December 2020. Simultaneously, blood specimens were collected from 307 healthy children who underwent routine pediatric outpatient examinations during the same period. The case-control matching function of SPSS 25.0 statistical software was used to match the children in a 1:1 ratio based on the same birth year and sex. Finally, a total of 84 children with OI (OI group) and 84 healthy children (control group) were included as research subjects. This study was approved by the Medical Ethics Committee of the Lanzhou University Second Hospital [2018A-002]. The original consent form for routine blood tests stated that leftover samples could be used for medical research, and this study does not involve commercial purposes or sensitive information.

### 2.2 Inclusion criteria

(1) Meet the diagnostic criteria for VVS or POTS according to 2018 Chinese Pediatric Cardiology Society (CPCS) guideline for diagnosis and treatment of syncope in children and adolescents. In brief, a positive diagnosis of VVS is indicated by the occurrence of syncope or presyncopal symptoms (dizziness, vertigo, headache, chest tightness, palpitations, nausea, vomiting, pallor, hyperhidrosis, blurred vision, hearing loss, or abdominal pain) during the HUTT, along with any of the following criteria: (1) SBP ≤80 mmHg, DBP ≤50 mmHg, or a mean arterial pressure decrease of ≥25%; (2) HR falling below age-specific thresholds: <75 bpm (ages 4–6), <65 bpm (ages 7–8), and <60 bpm (ages >8); (3) ECG showing sinus arrest or junctional premature beats; (4) atrioventricular block or cardiac arrest ≥3 s. POTS is diagnosed during HUTT or standing test when: (1) HR is normal in the supine position; (2) within 10 min, HR increases by ≥40 bpm, or reaches ≥130 bpm (ages 6–12) or ≥125 bpm (ages 13–18); (3) no orthostatic hypotension (BP drop >20/10 mmHg) (16);
(2) Complete clinical data: for the OI group, complete HUT/HUTT test reports and a clear diagnosis should be provided. These patients must be newly diagnosed and have not received any prior treatment. For the healthy control group, only children who have undergone physical examinations at a health check-up center and show no apparent abnormalities will be included. All participants are permanent residents of Lanzhou City and are aged between 5 and 15 years.
(3) Written informed consent from the guardians of the children.

### 2.3 Exclusion criteria

(1) Children with a previous diagnosis of any autonomic dysfunction (including dysautonomia, vagal nerve dysfunction), congenital heart disease, arrhythmias (such as ventricular premature beats, tachycardia, etc.), type 1 diabetes, thyroid disorders (hyperthyroidism or hypothyroidism), or diseases such as myasthenia, poliomyelitis, or chronic fatigue syndrome.
(2) Children who are currently using or have a history of long-term use of RAAS inhibitors, such as angiotensin-converting enzyme inhibitors (ACEIs), angiotensin receptor blockers (ARBs), aldosterone antagonists, direct renin inhibitors (DRIs), and central renin inhibitors, as well as β-blockers, diuretics, antidepressants, corticosteroids, or vitamin D supplements.

### 2.4 Study methods

Grouping:

1. Orthostatic intolerance group and control group.
2. Based on the results of HUT/HUTT, the orthostatic intolerance group was further divided into vasovagal syncope group and postural tachycardia syndrome group.
3. According to the vitamin D diagnostic criteria of the Chinese Pediatric Society, serum 25(OH)D levels were categorized as sufficient (>50 nmol/L or >20 ng/ml), insufficient (37.5–50 nmol/L or 15–20 ng/ml), deficient (<37.5 nmol/L or <15 ng/ml), and severely deficient (<12.5 nmol/L or 5 ng/ml) (17).

### 2.5 Detection indicators and methods

Enzyme-linked immunosorbent assay was used to measure the levels of ACE2, Ang(1-7) enzyme, 25-hydroxylase, 1α-hydroxylase, and 24-hydroxylase in human serum, provided by Shanghai Jianglai Biotechnology Co., Ltd. Serum 25(OH)D concentrations were measured using the Roche cobas 6,000 fully automatic electrochemiluminescence immunoassay analyzer.

### 2.6 Statistical methods

Data were analyzed using SPSS 25.0. Descriptive statistics were presented as counts (percentages) for categorical variables, and normally distributed continuous variables were expressed as means ± standard deviation (x ± s). Group comparisons were conducted using the chi-square test for categorical data, independent sample *t*-tests or corrected *t*-tests for normally distributed continuous data, and the Mann–Whitney *U*-test for non-normally distributed continuous data. One-way analysis of variance was used for group comparisons of normally distributed continuous data, with *post hoc* pairwise comparisons corrected using the LSD method. For non-normally distributed continuous data, the Kruskal–Wallis *H*-test was employed, with *post hoc* pairwise comparisons corrected using the Bonferroni method. Binary logistic regression analysis was conducted to identify factors related to the onset of orthostatic intolerance. Receiver operating characteristic curve analysis was performed to evaluate the diagnostic predictive value of 25(OH)D for orthostatic intolerance. A *p*-value less than 0.05 was considered statistically significant.

## 3 Result

### 3.1 Baseline data of orthostatic intolerance patients and controls

The OI group had an average age of 10.89 ± 1.92 years, with 47 boys and 37 girls. The median age for boys was 11.0 (2.0) years, and for girls, it was 11.0 (2) years. The control group had an average age of 10.43 ± 2.08 years, with 47 boys and 37 girls. The median age for boys was 10.0 (3.0) years, and for girls, it was 11.0 (4.0) years. There were no statistically significant differences in sex and age between the two groups (*P* > 0.05).

In the OI subgroup, POTS accounted for 39.2% (33/84) and VVS accounted for 60.7% (51/84). The POTS group comprises 19 boys (57.6%) with an average age of 10.58 ± 1.90 years and 14 girls (42.4%) with an average age of 11.79 ± 1.12 years, resulting in an overall average age of 11.09 ± 1.70 years. The VVS group includes 28 boys (54.9%) with an average age of 10.93 ± 1.76 years and 23 girls (45.1%) with an average age of 10.57 ± 2.39 years, with a combined average age of 10.76 ± 2.06 years. The healthy control group consists of 47 boys (56.0%) with an average age of 10.17 ± 1.90 years and 37 girls (44.0%) with an average age of 10.76 ± 2.27 years, resulting in an overall average age of 10.43 ± 2.08 years (Supplementary Table S1).

### 3.2 Comparison of ACE2 and Ang(1-7) levels in orthostatic intolerance group and its subgroups with the control group

**Figure 1:**
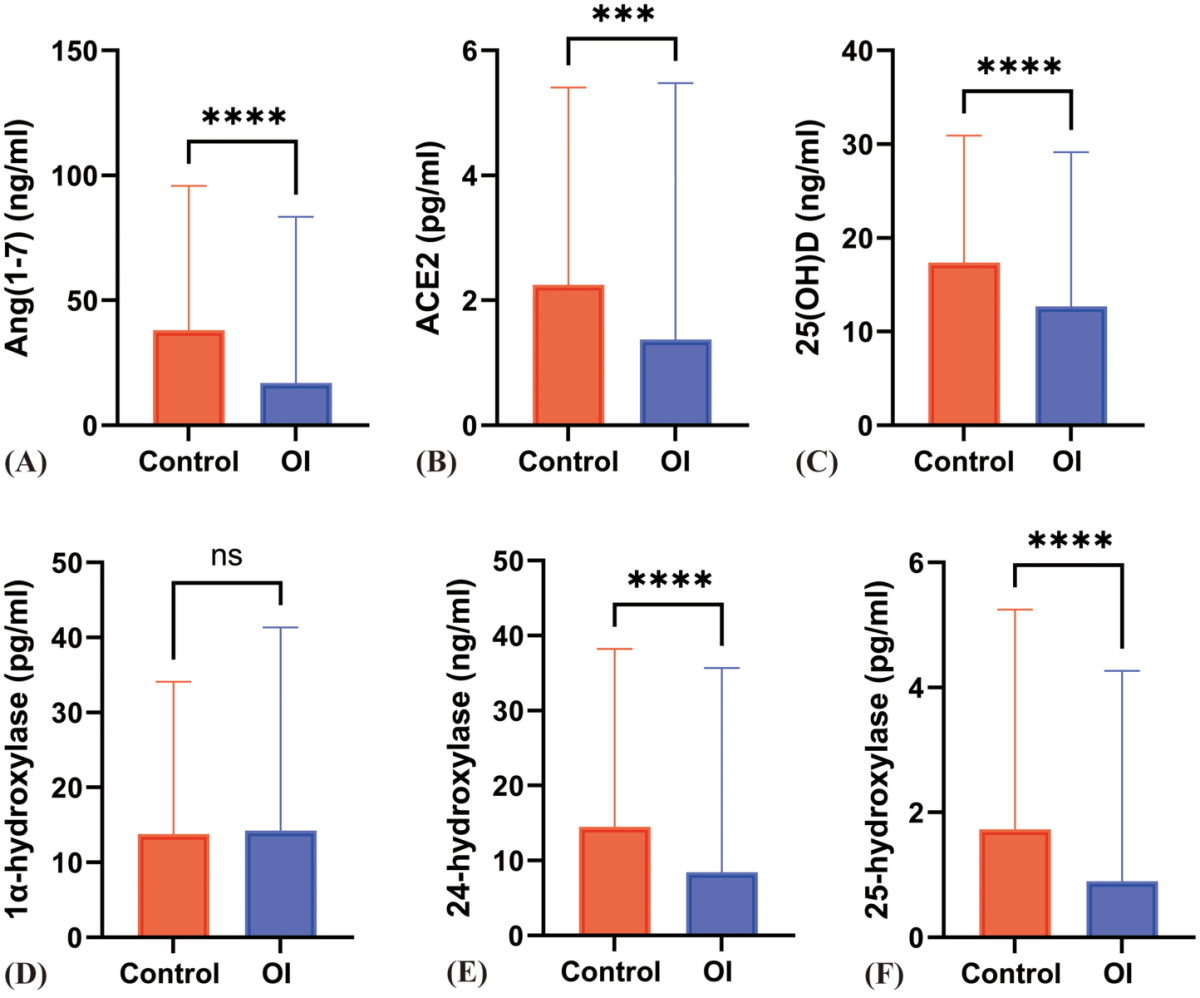
Differences in various indicators between the OI group and the control group. **(A)** to **(F)** respectively represent the differences in Ang(1–7), ACE2, 25(OH)D, 1α-hydroxylase, 24-hydroxylase, and 25-hydroxylase levels between the Control and OI groups. *** indicates *P* < 0.0005, **** indicates *P* < 0.0001, and ns indicates *P* > 0.05.

In the OI group, the serum ACE2 levels [1.46 (1.80) vs. 2.41 ± 1.33 pg/ml] (*z* = −3.66, *P* < 0.001) and Ang(1-7) levels [16.98 (21.89) vs. 38.07 (38.20) ng/ml, *z* = −6.43, *p* < 0.001] were significantly lower compared to the control group, with statistically significant differences (Figures 1A,B).

**Figure 2:**
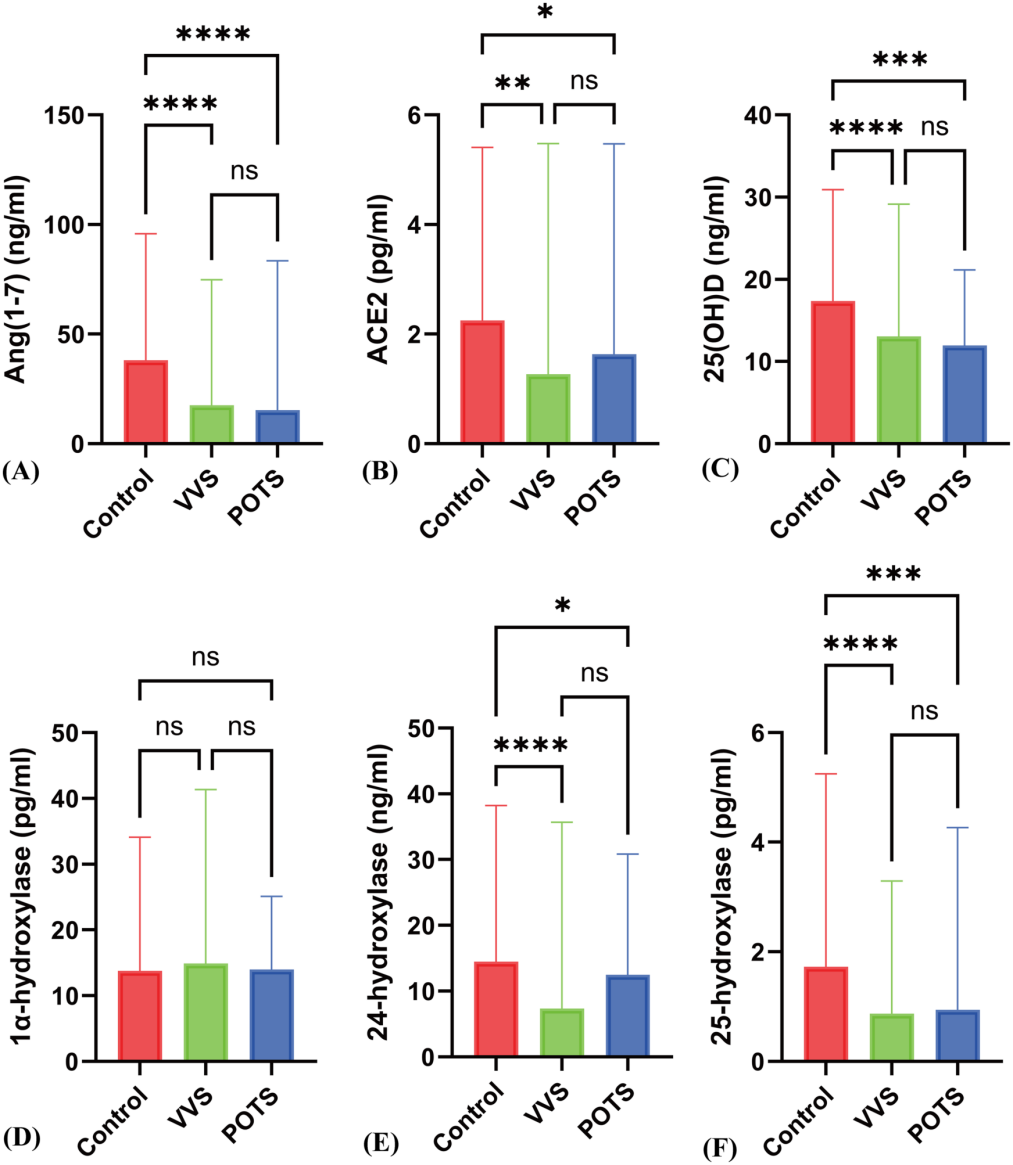
Differences in various indicators between the POTS group, VVS group, and control group. **(A)** to **(F)** respectively represent the differences in Ang(1–7), ACE2, 25(OH)D, 1α-hydroxylase, 24-hydroxylase, and 25-hydroxylase levels among the Control, VVS, and POTS groups. * indicates *P* < 0.01, ** indicates *P* < 0.005, *** indicates *P* < 0.0005, **** indicates *P* < 0.0001, and ns indicates *P* > 0.05.

In the VVS group, the serum ACE2 levels [2.38 ± 1.30 ± 1.47 vs. 1.27 (1.45) pg/ml] and POTS group serum ACE2 levels [2.38 ± 1.30 vs. 1.93 (2.78) pg/ml] were both significantly decreased compared to the control group, with statistically significant differences (*P* < 0.05). The VVS group, Ang(1-7) levels [38.07 (38.20) vs. 17.52 (18.54) ng/ml] and POTS group Ang(1-7) levels [38.07 (38.20) vs. 15.30 (34.99) ng/ml] were significantly lower than those in the control group, with statistically significant differences (*P* < 0.05) (Figures 2A,B).

### 3.3 Comparison of serum ACE2 and Ang(1-7) levels between orthostatic intolerance group and control group by sex

**Figure 3:**
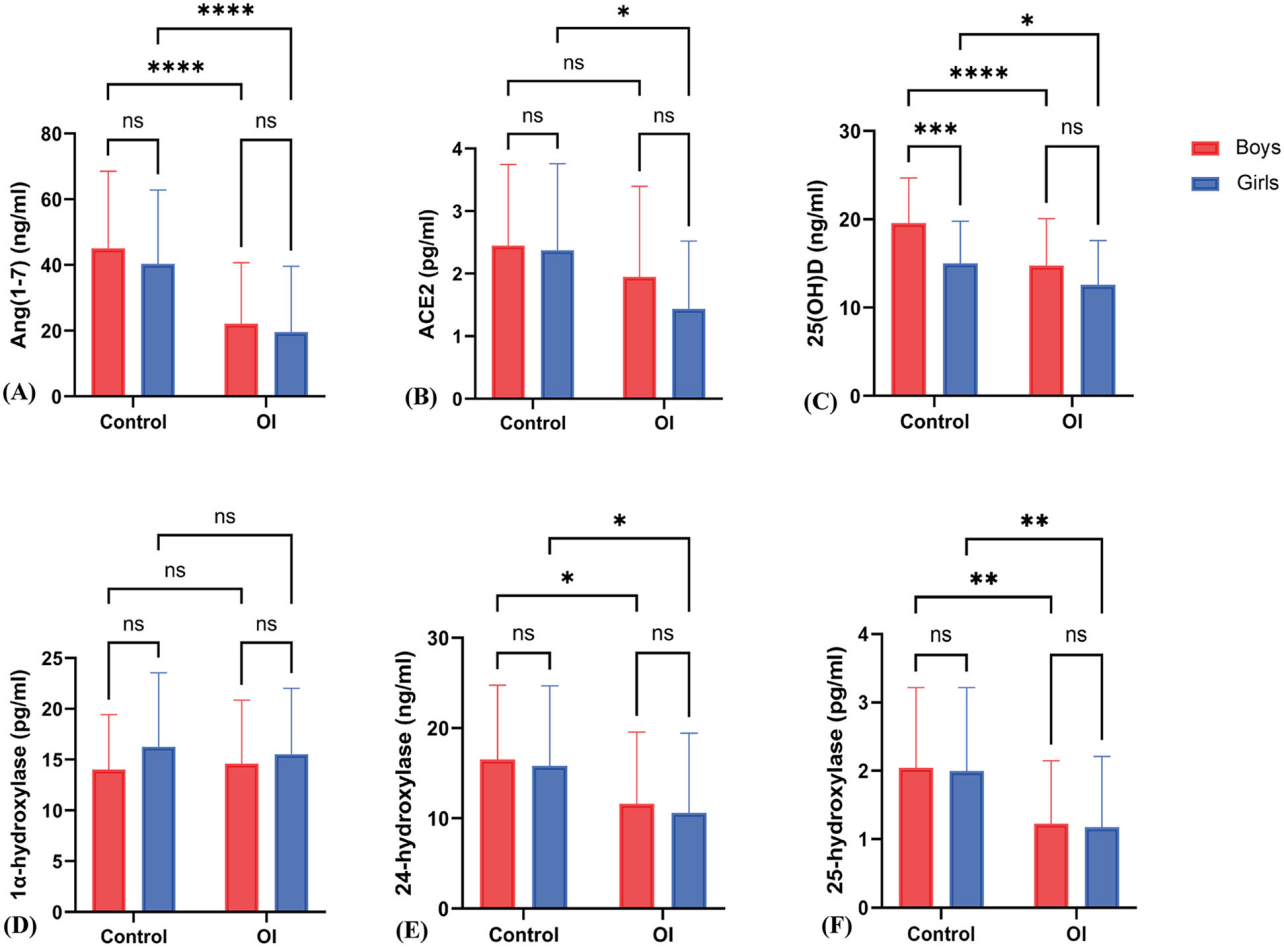
Sex-specific differences in various indicators between the OI group and the control group. **(A)** to **(F)** respectively represent the differences in Ang(1–7), ACE2, 25(OH)D, 1α-hydroxylase, 24-hydroxylase, and 25-hydroxylase levels between boys and girls in the Control and OI groups. * indicates *P* < 0.01, ** indicates *P* < 0.005, *** indicates *P* < 0.0005, **** indicates *P* < 0.0001, and ns indicates *P* > 0.05.

In control group girls, ACE2 levels were higher than in OI group girls {[2.37 ± 1.39 vs. 1.16 (1.46) pg/ml]} (*z* = −3.10, *P* = 0.002); Ang(1-7) levels in control group girls {[40.40 ± 22.45 vs. 14.12 (21.94) ng/ml]} were also higher than in OI group girls (*z* = −4.20, *P* < 0.001). In control group boys, ACE2 levels {[2.45 ± 1.30 vs. 1.56 (1.85) pg/ml]} were higher than in OI group boys (*z* = −2.14, *P* = 0.045); Ang(1-7) levels in control group boys {[45.06 ± 23.53 vs. 18.48 (25.72) ng/ml]} were also higher than in OI group boys (*z* = −4.79, *P* < 0.001) (Figures 3A,B).

There was no statistically significant difference in ACE2 levels [2.37 ± 1.39 vs. 2.45 ± 1.30 pg/ml] between control group girls and boys (*t* = 0.13, *P* = 0.96); there was also no statistically significant difference in Ang(1-7) levels [40.40 ± 22.45 vs. 45.06 ± 23.53 ng/ml] between control group girls and boys (*z* = −0.92, *P* = 0.36). In OI group boys and girls, there were no statistically significant differences in ACE2 levels [1.56 (1.85) vs. 1.16 (1.46) pg/ml] (*z* = −1.15, *P* = 0.25) and Ang(1-7) levels [18.48 (25.72) vs. 14.12 (21.94) ng/ml] (*z* = −1.21, *P* = 0.23) (Figures 3A,B).

### 3.4 Comparison of serum 25(OH)D and related hydroxylase levels between orthostatic intolerance and control groups

**Figure 4:**
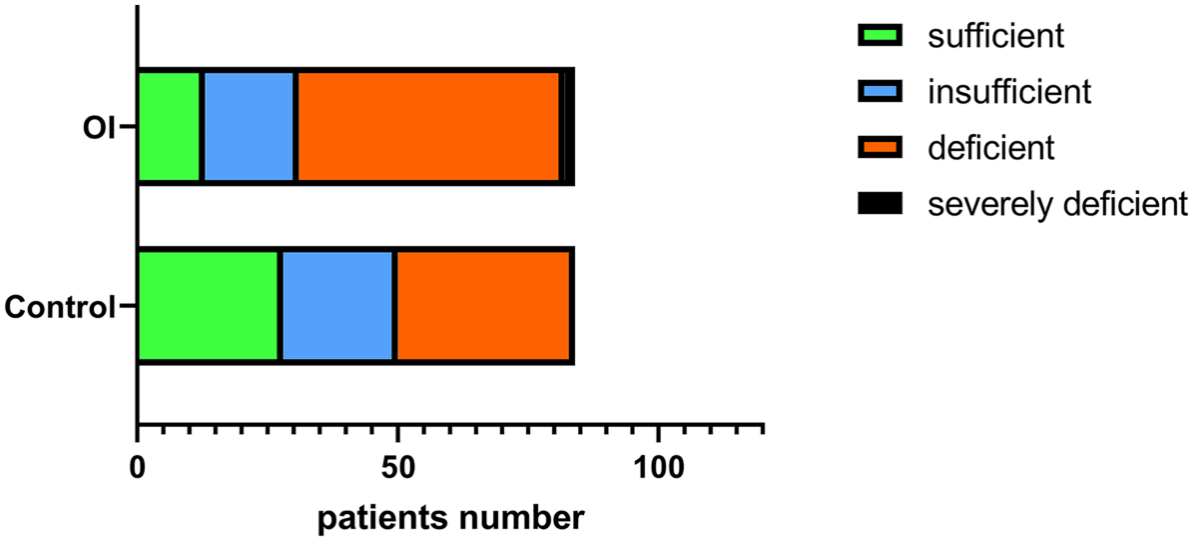
Stratified analysis of serum vitamin D levels in the OI group and the control group.

The serum levels of 25(OH)D, 24-hydroxylase, and 25-hydroxylase in the OI group were significantly lower than those in the control group (*P* < 0.05). However, there was no significant difference in the level of 1α-hydroxylase (*P* = 0.95) (Figures 1C–F). The proportion of individuals with 25(OH)D deficiency in the orthostatic intolerance group is higher, in the orthostatic intolerance group, the rates of inadequacy, deficiency, or severe deficiency of vitamin D are as high as 84.5% (Figure 4).

### 3.5 Comparison of serum 25(OH)D and related hydroxylase levels between different orthostatic intolerance subtypes and the control group

Compared to the control group, patients with VVS and POTS showed a significant decrease in serum 25(OH)D levels [17.56 ± 5.45 vs. 12.91 (8.52), 17.56 ± 5.45 vs. 13.10 ± 4.45 ng/ml] (*P* < 0.05). The levels of 24-hydroxylase {[16.21 ± 8.48 vs. 12.38 ± 7.90, 16.21 ± 8.48 vs. 7.36 (11.15)]} were also significantly decreased (*P* < 0.001), and the levels of 25-hydroxylase [1.73 (1.73) vs. 0.87 (0.69), 1.73 (1.73) vs. 0.94 (1.56) pg/ml] showed a significant reduction (*P* < 0.001). However, there was no statistical difference in 1α-hydroxylase levels [15.00 ± 6.37 vs. 15.52 ± 7.18 vs. 14.20 ± 4.82 pg/ml] (*z* = 0.604, *P* = 0.57). Additionally, there were no significant differences between POTS and VVS patients in 25(OH)D, 24-hydroxylase, and 25-hydroxylase levels (Figures 1C–F; Figures 2C–F).

### 3.6 Comparison of serum 25(OH)D and related hydroxylase levels in different sex between the orthostatic intolerance group and control group

In the control group, girls exhibited higher levels in 25(OH)D (15.01 ± 4.79 vs. 12.56 ± 5.03 ng/ml) (*t* = 2.15, *P* = 0.035), 24-hydroxylase [15.82 ± 8.88 vs. 7.41 (12.37) pg/ml] (*z* = −2.92, *P* = 0.007), and 25-hydroxylase [2.00 ± 1.22 vs. 0.78 (1.00)] (*z* = −3.40, *P* = 0.001) compared to girls in the OI group. However, there was no significant difference in 1α-hydroxylase levels [16.25 ± 7.30 vs. 14.57 (7.13) pg/ml] (*t* = −0.524, *P* = 0.60) (Figures 3C–F).

In boys from the control group, higher levels were observed in 25(OH)D (19.56 ± 5.13 vs. 14.74 ± 5.34 ng/ml) (*t* = 4.49, *P* < 0.001), 24-hydroxylase [16.52 ± 8.24 vs. 8.93 (11.01) pg/ml] (*z* = −2.88, *P* = 0.004), and 25-hydroxylase [1.73 (1.90) vs. 0.93 (0.90)] (*z* = −4.00, *P* < 0.001) compared to boys in the OI group. However, no significant differences were found in 1α-hydroxylase levels (14.01 ± 5.40 vs. 14.60 ± 6.27 pg/ml) (*t* = −0.482, *P* = 0.631).

Furthermore, boys in the control group had higher levels of 25(OH)D (19.58 ± 5.12 vs. 15.01 ± 4.79 ng/ml) compared to girls in the control group, with statistical significance (*t* = −4.180, *P* < 0.001). On the other hand, for OI group, there are no significant differences were observed in 25(OH)D, 1α-hydroxylase level, 24-hydroxylase levels, and 25-hydroxylase levels between boys and girls (Figures 3C–F).

### 3.7 Binary logistics regression analysis of factors related to orthostatic intolerance

Using sex, age, Ang(1-7), ACE2, 25(OH)D, 1α-hydroxylase, 24-hydroxylase, and 25-hydroxylase as independent variables, and the presence of OI as the dependent variable, binary logistic regression (forward LR method) analysis was performed to obtain the final model coefficients.

Logistic regression analysis suggested that 25(OH)D, 25-hydroxylase and Ang(1-7) were negatively correlated with the incidence of OI. each unit decrease in these indicators increased the corresponding morbidity rates by 10.4% (OR = 0.896, 95%CI: 0.826–0.972, *P* = 0.009), 81.9% (OR = 0.181, 95%CI: 0.054–0.604, *P* = 0.005), and 13.7% (OR = 0.863, 95%CI: 0.737–0.899, *P* < 0.001), respectively. The level of ACE was positively correlated with the occurrence of OI, and the incidence of OI increased 16.8 times for every unit increase in ACE2 (OR = 16.801, 95%CI: 5.385–52.42, *P* < 0.001) (Table 1).

**Table 1:** Binary logistics regression analysis of factors related to orthostatic intolerance.

Variables	β	S.E.	Wald	*P*	OR	95% C.I.	
						Lower	Upper
Constant	2.952	0.74	15.939	<0.001	19.152		
25(OH)D	−0.109	0.042	6.923	0.009	0.896	0.826	0.972
Ang(1-7)	-0.148	0.032	21.17	<0.001	0.863	0.81	0.919
ACE2	2.821	0.581	23.618	<0.001	16.801	5.385	52.42
25 hydroxylase	-1.709	0.614	7.735	0.005	0.181	0.054	0.604

Hosmer and Lemeshow test: χ^2^ = 4.95 *P* = 0.73.

Omnibus tests of model coefficients: χ^2^ = 95.36 *P* < 0.001.

### 3.8 Diagnostic value of 25(OH)D, 24-hydroxylase, 25-hydroxylase, and 1α-hydroxylase for orthostatic intolerance

**Figure 5:**
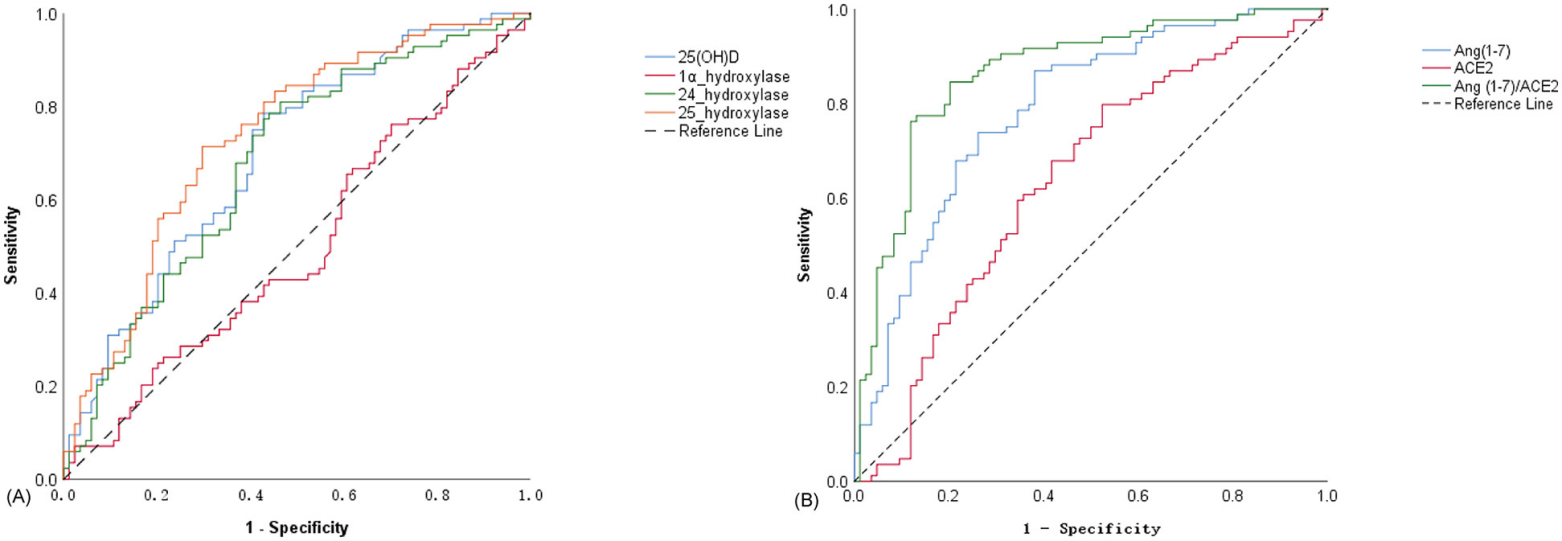
ROC curves for the diagnostic value of different indicators in OI. **(A)** ROC curve of 25(OH)D, 1α-hydroxylase, 24-hydroxylase and 25-hydroxylase in the diagnosis of OI; **(B)** ROC curves of Ang(1–7), ACE2 and Ang(1–7)/ACE2 ratio in the diagnosis of OI.

The diagnostic value of 25(OH)D, 24-hydroxylase, 25-hydroxylase, and 1α-hydroxylase for OI was assessed using receiver operating characteristic curves. Using 13.205 ng/ml as the threshold for 25(OH)D, the sensitivity and specificity for diagnosing orthostatic intolerance were 78.6% and 57.1%, respectively. For 24-hydroxylase, with a threshold of 9.17 pg/ml, the sensitivity was 81.0%, and the specificity was 53.6%. using 1.24 pg/ml as the threshold for 25-hydroxylase, the sensitivity and specificity for diagnosing orthostatic intolerance were 71.4% and 70.2%, as shown in Table 2 and Figure 5A.

**Table 2:** Diagnostic value of 25(OH)D, 24-hydroxylase and 25-hydroxylase for OI.

Variables	Area under the curve	95% C.I.	Std. Errora	Diagnostic threshold	Sensitivity	Specificity	Youden's index	*P*
25(OH)D	0.698	0.619–0.777	0.040	13.205	0.786	0.571	0.357	<0.001
25 hydroxylase	0.679	0.598–0.760	0.041	1.23606	0.714	0.702	0.416	<0.001
24 hydroxylase	0.735	0.659–0.810	0.039	9.1709	0.81	0.536	0.346	<0.001

### 3.9 Diagnostic value of ACE2, Ang(1-7), and the ratio of Ang(1-7) to ACE2 for orthostatic intolerance

Due to the close correlation between Ang(1-7) and ACE2, considering them together better reflects their regulatory effect on the ACE2-Ang(1-7)-Mas Axis. The diagnostic value of ACE2, Ang(1-7), and the ratio of Ang(1-7) to ACE2 for orthostatic intolerance was assessed using receiver operating characteristic curves curves. Using a threshold of 1.29 pg/ml for ACE2, the sensitivity and specificity for diagnosing orthostatic intolerance were 79.8% and 47.6%, respectively. For Ang(1-7), with a threshold of 19.39 ng/ml, the sensitivity was 86.9%, and the specificity was 61.9%. Using a threshold of 14.89 pg/ml for the ratio of Ang(1-7) to ACE2, the sensitivity and specificity for diagnosing orthostatic intolerance were 84.5% and 79.8%, respectively. Among these indicators, Ang(1-7) exhibited the largest area under the curve, indicating the highest diagnostic sensitivity. In contrast, the ratio of Ang(1-7) to ACE2 had the highest Youden's Index, reflecting the strongest diagnostic specificity, as shown in Table 3 and Figure 5B.

**Table 3:** Diagnostic value of ACE2, Ang(1-7), and the ratio of Ang(1-7) to ACE2 for OI.

Variables	Area Under the Curve	95% C.I.	Std. Errora	Diagnostic threshold	Sensitivity	Specificity	Youden's indx	*P*
Ang1-7	0.787	0.719–0.856	0.035	19.385	0.869	0.619	0.488	<0.001
ACE2	0.635	0.550–0.720	0.043	1.289	0.798	0.476	0.274	0.002
Ang(1-7)/ACE2	0.865	0.808–0.921	0.029	14.892	0.845	0.798	0.643	<0.001

## 4 Discussion

### 4.1 The widespread vitamin D deficiency may contribute to orthostatic intolerance in children

25(OH)D is the primary circulating form of vitamin D in the body, characterized by lower biological activity, a longer half-life, and higher sensitivity. It serves as the main storage form of vitamin D in the body and is currently an important biomarker for measuring serum vitamin D levels in clinical practice (18, 19). Our study found that vitamin D deficiency is present in both healthy children and children with OI, with approximately 84.5% of OI patients having vitamin D levels below the normal threshold (<20 ng/ml). This finding is consistent with epidemiological surveys conducted in our country, which indicate that vitamin D deficiency and insufficiency are common among children, and the prevalence of deficiency tends to increase with age (20). Our study data derived from the Lanzhou region, suggest that vitamin D deficiency in children may be related to the region's high latitude and insufficient sunlight exposure. Moreover, the vitamin D levels in OI children were generally lower than those of sex-matched healthy children, and this deficiency or insufficiency was observed in various types of OI, including VVS and POTS. This indicates that vitamin D deficiency might be a widespread issue among OI children. Vitamin D deficiency could contribute to a range of physiological dysfunctions by impairing its regulatory effect on the autonomic nervous system, such as inhibiting sympathetic nerve activity and enhancing parasympathetic tone (3, 4, 7, 21). Furthermore, vitamin D influences the synthesis of nitric oxide (NO) by regulating the activity of endothelial nitric oxide synthase (NOS), which in turn modulates smooth muscle contraction (22, 23). Vitamin D deficiency may be a cause of orthostatic intolerance, as the incidence of orthostatic intolerance significantly increases when vitamin D is lacking, estimated to be twice as high as that of normal adolescents lacking vitamin D (30% vs. 14%) (24, 25). In Anthony's study, it was also found that 50% of POTS patients met the criteria for vitamin D insufficiency, and an additional 7% met the criteria for vitamin D deficiency, totaling 58% of POTS patients with abnormal serum vitamin D levels (26). A single-center retrospective study on the correlation between vasodilation and vasovagal syncope revealed a significant decrease in vitamin D levels in fainting patients. No significant differences were observed among vasovagal syncope subgroups, but low vitamin D levels were significantly correlated with syncope (27). Therefore, vitamin D deficiency may lead to autonomic dysfunction and vascular impairment, providing a potential pathological basis for the development of OI.

### 4.2 Role of vitamin D metabolizing enzymes in orthostatic intolerance

25-hydroxylase and 1α-hydroxylase are the key enzymes involved in the synthesis of the active form of vitamin D, 1,25(OH)2D. A deficiency in the activity of these enzymes can result in insufficient production of active vitamin D due to impaired synthesis. Conversely, excessive activity of 24-hydroxylase, which is responsible for the degradation of both 1,25(OH)2D and 25(OH)D, can lead to an accelerated breakdown of active vitamin D, resulting in deficiency (5, 6). The endothelial function mediated by vitamin D is closely linked to the VDR and 1α-hydroxylase (28). 1α-hydroxylase is predominantly located in the kidneys but is also present in extrarenal tissues, including immune cells and keratinocytes. This enzyme serves as the critical rate-limiting factor in the synthesis of 1,25(OH)2D, playing a central role in regulating the body's levels of active vitamin D (29). Mice deficient in 1α-hydroxylase show increased RAAS activity, leading to hypertension and other symptoms due to elevated plasma renin. This activity can be downregulated by the administration of 1,25(OH)2D (13). In cases of 1α-hydroxylase deficiency in postural orthostatic tachycardia syndrome (POTS), serum 1,25(OH)2D levels are lower than normal. Supplementation with calcitriol significantly improves orthostatic intolerance and symptoms such as palpitations, suggesting that 1α-hydroxylase plays a regulatory role in conditions like OI (30).

Our study found that the levels of 25-hydroxylase and 24-hydroxylase were significantly lower in OI children compared to healthy controls, with statistically significant differences. However, no significant differences were observed in the levels of 1α-hydroxylase. These findings indicate that the decreased vitamin D levels in OI children cannot be solely attributed to enhanced degradation. Instead, it may be primarily due to insufficient concentrations or activity of the synthetic enzyme 25-hydroxylase.

### 4.3 Renin-angiotensin-aldosterone system dysregulation in orthostatic intolerance

Early studies have found that children with postural orthostatic tachycardia syndrome (POTS) exhibit reduced blood volume, yet plasma renin and aldosterone levels do not increase accordingly, leading to the concept of the “renin-aldosterone paradox” in these patients (31). This paradox suggests that there may be a dysfunction in the regulation of angiotensin II (Ang II), potentially due to excessive production of Ang II, reduced degradation, or abnormalities in the Ang II receptor AT1R. The presence of AT1R antibodies may also negatively modulate the action of Ang II (32). Furthermore, the “renin-aldosterone paradox” in POTS may be linked to a decrease in the activity of ACE2, an enzyme responsible for converting Ang II into Ang(1-7). When ACE2 activity is reduced, the ACE2-Ang(1-7)-Mas axis becomes dysregulated, leading to the loss of cardiovascular protective effects and potentially triggering syncope or pre-syncope symptoms (33). Similar RAAS dysfunction has also been reported in children with vasovagal syncope (VVS), characterized by slightly increased renin and Ang II levels, but decreased aldosterone levels (27). Current research suggests that the reduction in ACE2 activity may be linked to negative feedback mechanisms involving elevated Ang II and low blood volume. Additionally, ACE2 expression tends to decrease with age, and its activity is lower in males than in females (likely due to estrogen upregulating ACE2 expression and the fact that ACE2 is located on the X chromosome), which may help explain why children with osteogenesis imperfecta (OI) tend to be older, and why the prevalence of the condition is higher in females compared to males (34, 35). We observed that serum levels of ACE2 and Ang(1-7) were significantly lower in OI patients compared to the control group. This suppression was consistently seen in both VVS and POTS subtypes. We hypothesize that vitamin D may enhance ACE2 expression, promoting the conversion of Ang II to Ang(1-7), thereby mitigating the negative effects of Ang II accumulation. Furthermore, when the RAAS system in OI patients is severely dysregulated, it may exacerbate autonomic dysfunction, worsening pre-syncope or syncope symptoms.

Finally, the ratio of Ang(1-7) to ACE2 in OI patients, with a critical value of 14.892, demonstrated a sensitivity of 84.5% and a specificity of 79.8% for diagnosing OI. Therefore, further exploration of the Ang(1-7)/ACE2 ratio as a potential valuable biomarker for identifying OI in clinical practice is warranted.

### 4.4 Limitations of this study

This study has several limitations: First of all, it only measured plasma levels of 25(OH)D, 24-hydroxylase, 25-hydroxylase, ACE2, and Ang(1-7), without delving into the specific mechanisms of action of these indicators. Secondly, the study is a single-center, small sample research, and a larger sample size is needed for further validation. Finally, the causal relationships between the indicators were not explored, and it remains unclear whether vitamin D deficiency leads to reduced levels of 25-hydroxylase and ACE2 Ang(1-7), or whether low levels of 25-hydroxylase contribute to vitamin D deficiency, which requires further investigation.

## 4 Conclusion

In conclusion, children with OI exhibit a significant deficiency in 25(OH)D, which may be associated with a decrease in 25-hydroxylase activity. This, in turn, could lead to the suppression of the ACE2-Ang(1-7)-Mas axis, resulting in RAAS imbalance, autonomic dysfunction, and vascular abnormalities, ultimately triggering OI symptoms. Vitamin D supplementation and strategies aimed at enhancing ACE2-Ang(1-7)-Mas axis activity may serve as potential therapeutic approaches. Furthermore, the ratio of Ang(1-7)/ACE2, as a potential diagnostic biomarker, warrants further investigation.

## Data Availability

The original contributions presented in the study are included in the article/Supplementary Material, further inquiries can be directed to the corresponding author.
