# Supplementary material for: Correlation between orthostatic intolerance in children and levels of ACE2-Ang(1-7)-Mas axis and vitamin D
Source: Front Pediatr. 2025 May 16;13:1585032. doi: 10.3389/fped.2025.1585032 (PMC12122540; doi:10.3389/fped.2025.1585032)
Supplement: Supplementary file 1 [file Table1.docx]

**Supplementary Table 1 Baseline characteristics of the patients in each group**

|  | Boys (n/%) | Girls (n/%) | Boys age | Girls age | Average age |
| --- | --- | --- | --- | --- | --- |
| POTS | 19(57.6%) | 14(42.4%) | 10.58±1.90 | 11.79±1.12 | 11.09±1.70 |
| VVS | 28(54.9%) | 23(45.1%) | 10.93±1.76 | 10.57±2.39 | 10.76±2.06 |
| Healthy control | 47(56.0%) | 37(44.0%) | 10.17±1.90 | 10.76±2.27 | 10.43±2.08 |
